# The effect of bilateral ultrasound-guided erector spinae plane block on postoperative pain control in idiopathic scoliosis patients undergoing posterior spine fusion surgery: study protocol of a randomized controlled trial

**DOI:** 10.1186/s13063-024-08331-2

**Published:** 2024-07-22

**Authors:** Jingchun Gao, Yi Ren, Dong Guo

**Affiliations:** 1 Department of Orthopedics, Beijing Children’s Hospital, Capital Medical University, National Center for Children’s Health, Beijing, China; 2 Department of Anesthesiology, Beijing Children’s Hospital, Capital Medical University, National Center for Children’s Health, Beijing, China

**Keywords:** Erector spinae plane block, Postoperative pain, Posterior spinal fusion, Adolescent idiopathic scoliosis

## Abstract

**Background:**

Posterior spinal fusion (PSF) for the correction of idiopathic scoliosis is associated with severe postoperative pain. Erector spinae plane block (ESPB) has been proposed to provide analgesia and reduce opioid consumption. We aimed to investigate the effect of bilateral ultrasound-guided single-shot ESPB on postoperative analgesia in pediatric patients undergoing PSF.

**Methods:**

This double-blinded, randomized controlled trial will enroll 74 AIS patients undergoing elective PSF. Participants will be assigned to the ESPB group or control group at a 1:1 ratio. Patients in the ESPB group will receive ultrasound-guided bilateral ESPB preoperatively, and patients in the control group received sham ESPB using normal saline. The primary joint endpoints are the area under the curve (AUC) of numerical rating scale (NRS) score and opioid consumption in postoperative 24 h. The secondary endpoints are numerical rating scale (NRS) score and opioid consumption at postoperative 0.5, 3, 6, 9, 12, 24, 36, and 48 h, rescue analgesia, recovery outcomes, and adverse events.

**Discussion:**

At present, studies investigating the effect of ESPB on pediatric patients are still needed. This study focuses on the effect of ESPB on pediatric patients undergoing PSF on postoperative pain control and intends to provide a new strategy of multimodal analgesia management for major spine surgery.

**Trial registration:**

Chinese Clinical Trial Registry ChiCTR2300074505. Registered on August 8, 2023.

**Supplementary Information:**

The online version contains supplementary material available at 10.1186/s13063-024-08331-2.

## Introduction

Adolescent idiopathic scoliosis (AIS) is one of the most common type of scoliosis, with a global prevalence of 1–4% [1]. AIS can occur in children under 3 years of age but usually affects patients aged 10 years of age to maturity [2]. It is defined by a three-dimensional progressive deformity of the spine caused by a complex interplay of genetic, internal, and environmental factors [3]. Severe deformity affects the physical and mental health of young people [1] and curves greater than 40° require surgery [1]. To correct the deformity, standard posterior spinal fusion (PSF) is the surgical treatment for AIS patients [4]. Due to the invasiveness of the procedure, long-lasting reflex muscle spasms, and deep somatic pain caused by tissue trauma, PSF is associated with severe and excruciating postoperative pain [5]. Inadequate pain control is associated with poor outcomes, late mobilization, and hospital discharge [6]. Moreover, younger age is thought to be an independent factor of increased pain after surgery [7], and therefore in many pediatric patients, strong doses of opioids need to be administered for postoperatively pain control [8]. Thus, a multimodal analgesia approach combined with regional blockage is important to optimize pain control and minimize adverse effects from opioids after scoliosis surgery.

Erector spinae plane block (ESPB) is a newly developed regional anesthesia originally reported for thoracoabdominal analgesia. The local anesthetic (LA) is injected beneath the erector spinae muscle and superficial to the vertebral transverse process and is expected to block not only the ventral rami of spinal nerves [9] but also the dorsal rami innervating the back [10–12] and, depending on dermatomal level placement and volume injected, lead to a sensory blockade involving dermatomes from T1 to sacral dermatomes [9]. The most plausible mechanism of ESPB currently is the spreading of LA on nerves passing within or contiguous with the erector spinae muscle through [13].

In practice, the transverse process can be the ultrasonographic landmark, which makes ESPB easy to operate, and the endpoint for the needle is away from the pleura and neuraxial structures, leading to fewer risks to nearby tissue and structures than neuraxial techniques [14]. As an effective postoperative analgesia technique, ESPB has now been used in a broad range of surgical interventions [15–18] and provides a superior analgesic effect [19,20] and reduced opioid consumption during the first 24 h operatively compared with systemic analgesia [21,22]. However, ESPB is placed mostly in adult patients (90.5%) [23]. Apart from case reports [24] and retrospective studies [25], its utility in pediatric patients remains unclear [26].

Therefore, we aimed to evaluate preoperative ultrasound-guided single-shot ESPB versus a control group (no block) in AIS patients undergoing PSF regarding postoperative pain score and dosage of opioid used.

## Material and methods

### Objective

The primary hypothesis is that ESPB reduces pain score and opioid consumption within 24 h postoperatively for AIS patients after PSF surgery compared with patients who received no block.

### Study design

**Fig. 1:**
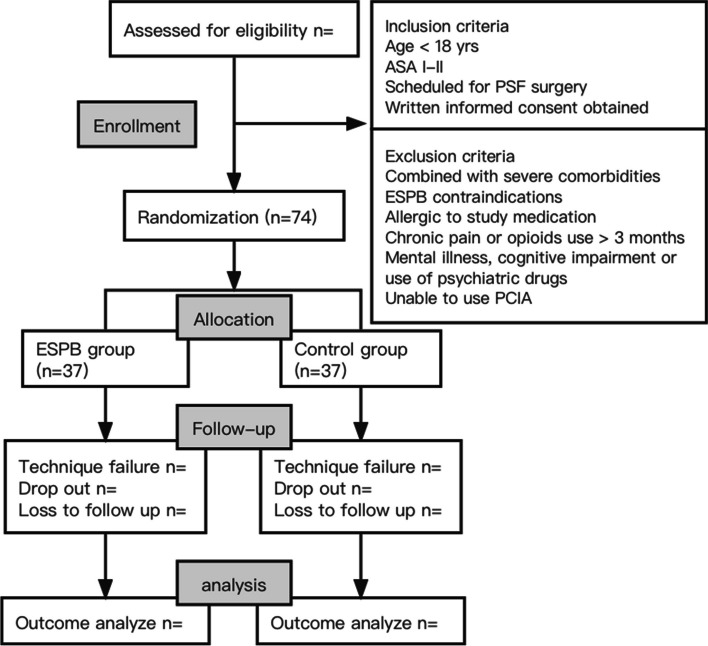
Flowchart of the study design. Abbreviations: ASA, American Society of Anesthesiologists; BMI, body mass index; ESPB, erector spinae block; PCIA, parent-controlled intravenous analgesia; PSF, posterior spinal fusion

This single-center, double-blinded, exploratory randomized controlled trial was approved by the Clinical Research Ethics Committee of Beijing Children’s Hospital ([2023]-E-064-Y), and its design has been completed in strict accordance with the SPIRIT 2013 statement. This study was prospectively registered before patient enrollment at https://www.chictr.org.cn (registration number: ChiCTR2300074505, principal investigator: Dong Guo) on August 8, 2023. Written informed consent will be obtained by a research assistant from the participants or their legal guardians in the case of children under 16 prior to study commencement. The findings will be disseminated through peer-reviewed publication, conference presentation, and social media to increase topic area knowledge and inform future research among researchers and healthcare professionals. This protocol adheres to the applicable Consolidated Standards of Reporting Trials (CONSORT) guidelines. The flowchart diagram of the study is presented in Fig. 1, and the SPIRIT figure of enrollment, interventions, and assessments is illustrated in Table 1.

**Table 1:** Trial schedule of enrollment, interventions, and assessments

**Time point**	**Study period**											
	**Enrollment**	**Allocation**	**Post-allocation**									**Close-out**
	− 1 D	0	PRE	POS 0.5 h	POS 3 h	POS 6 h	POS 9 h	POS 12 h	POS 24 h	POS 36 h	POS 48 h	Discharged day
**Enrollment**												
Eligibility screen	X											
Informed consent	X											
Allocation		X										
**Interventions**												
ESPB			X									
No block			X									
**Assessments**												
PCA boluses					X	X	X	X	X	X	X	
NRS score				X	X	X	X	X	X	X	X	
Postoperative complication				X	X	X	X	X	X	X	X	X
Mobilization				X	X	X	X	X	X	X	X	X
Gastrointestinal function				X	X	X	X	X	X	X	X	X
QoR-15 score								X		X	X	
Length of stay												X
Total cost												X

*PRE* preoperative, *POS* postoperative, *ESPB* erector spinae plane block, *NRS* numeric rating scale, *QoR-15 score* quality of recovery-15

### Participants

We include patients aged less than 18 years undergoing elective PIS with an American Society of Anesthesiologists (ASA) physical status of I to II. The exclusion criteria are as follows:

1. Severe comorbidities such as cardiac insufficiency and liver or renal dysfunction;
2. contraindications of ESPB: coagulation abnormality, hemorrhagic diseases, puncture site infection, and preexisting neurological deficits;
3. Allergy to LA or other study medication;
4. Chronic pain characterized by preoperative opioid use for more than 3 months;
5. Cognitive impairment or mental illness rendering the patient unable to cooperate in the pain assessment; and
6. Inability of the guardian to use a postoperative analgesia pump.

### Recruitment

The participants will be enrolled at our institution, which is a national scoliosis diagnosis and treatment center serving more than 200 children with scoliosis each year. Therefore, we used a large patient population as the basis of this trial. In addition, the following methods and strategies were used to ensure that sufficient participants were recruited: determining the type of subjects who were suitable and ensuring that the recruitment criteria were clear and specific; establishing partnerships with local healthcare providers, community organizations, and physicians to promote the trial and attract suitable patients to participate; posting information about the trial through medical journals, hospital websites, media, forums, the internet, and other channels to seek participants; and strictly complying with the rules of the ethics committee and laws and regulations to ensure the safety and rights of participants during the recruitment process.

### Randomization and blinding

Patients will be screened the day before surgery by the investigator. After assignments, the patients will be randomized on the morning of the day of surgery to the ESPB group or the control group at a 1:1 ratio according to a computer-generated sequence of random numbers generated by a third-party statistician not otherwise involved in the trial. Consecutively numbered, opaque, sealed envelopes will be used to conceal the sequence. Nurse anesthetists who will not participate in the follow-up visit will assign participants to the two groups. The LA given to patients in the ESPB group and the normal saline with the same volume given to patients in the control group will also be prepared by the anesthesia nurse according to the group allocation. The attending anesthesiologists who will implement the treatment, the patients and their guardians, and the investigator responsible for follow-up will be blinded to the randomization groups. The statistician will be unblinded to the allocation only after all the data have been collected, entered into the database, and cleaned, and the primary analysis has been completed. Emergency blinding is performed in the case of serious adverse events or patients needing urgent resuscitation so that the physician knows the patient grouping for resuscitation and reports it to the Principal Investigator.

### Intervention

#### General anesthesia and intraoperative pain management

All patients will undergo standardized monitoring. General anesthesia is induced with intravenous sufentanil 0.5 μg/kg, propofol 2.0 to 3.0 mg/kg, and cisatracurium 0.1 mg/kg, followed by propofol 0.1 to 0.2 mg kg^−1^ min^−1^ and sevoflurane 1–1.5 MAC for anesthesia maintenance to keep a bispectral index between 40 and 60. Remifentanil can be infused at 0.1 to 0.2 μg kg^−1^ min^−1^ if necessary. The anesthetic, vasopressors, fluid volume, and infusion speed are adjusted to maintain the hemodynamic parameters within 20% of the preoperative baseline values. After intubation, patients in the ESPB group will receive a bilateral ultrasound-guided erector spinae plane block using ropivacaine, and patients in the control group will receive a bilateral sham block and be turned to the prone position to begin the surgery. The intraoperative analgesic protocol consisted of intravenous administration of 5 mg dexamethasone before general anesthesia induction and 1 mg/kg dexmedetomidine 20 min before the end of surgery. Ibuprofen at 10 mg/kg (maximum: 400 mg) was diluted to a concentration less than 4 mg/ml using saline and administered intravenously at the beginning of surgery and thereafter every 6 h at the ward until 48 h postoperatively.

##### Surgical procedure

The surgeon will make a posterior midline incision and expose the spine gradually. After inserting the screws, rods are placed into the screws to correct the deformity. Local bone grafts are placed around the osteotomy sites. After incision suturing, the patients will be weaned from mechanical ventilation and extubated. All patients will be admitted to the post anesthesia care unit (PACU) and then discharged to the ward with a full evaluation of conscious state and vital signs.

##### Erector spinae plane block

For patients who undergo posterior spinal fusion that spans ≤ 5 vertebral segments, bilateral ESPB will be performed at the midpoint of the incision [25]. For patients with anticipated fusion of ≥ 6 segments, the planned incision will be divided into two segments, and bilateral ESPB will be performed at the midpoint of each segment.

A high-frequency (4 to 15 MHz) linear transducer (Labat SP; Wisonic, Shenzhen, China) will be used to identify the erector spinae muscle and transverse process at 2–3 cm lateral to the spinous process. After aseptic conditions, a 21-gauge block needle (5 cm, Hakko disposable monopolar nerve blockage needle, Hakko Co., Ltd., Nagano, Japan) will be inserted using the in-plane approach to access the transverse process. Once the correct location is confirmed, 0.3% ropivacaine in a volume of 0.5 ml/kg per side per level (maximum of 3 mg/kg) will be injected into the musculofascial plane between the erector spinae muscle and transverse process. The procedure is then repeated on the other side.

##### Postoperative analgesia

Electronic parent-controlled intravenous analgesia (PCIA) pumps (CPE-101, Fornia Medical Equipment Co., Ltd., Zhuhai, China) will be used for all participants in the two groups in 2 days after surgery. The PCIA regimen is sufentanil 0.04 μg/kg/ml and 0.1 mg/kg tropisetron in normal saline 200 ml. A bolus of 1 ml on demand with a lockout interval of 15 min was set, with no background infusion. The guardians of patients will be trained in advance to use the electronic pump. Acetaminophen 15 mg/kg orally will be given to patients in both groups four times daily (maximum dose of 2 g per 24 h). If the patient’s NRS pain score is >6 or if rescue analgesia is needed, dezocine (0.1 mg/kg, maximum dose of 5 mg) will be given.

### Endpoints

#### Primary endpoint

The primary outcome was a joint endpoint of area under the curve (AUC) of numerical rating scale (NRS) score over time and opioid consumption in postoperative 24 h. NRS score (0 = no pain and 10 = the worst pain) at rest was measured at 0.5 h, 3 h, 6 h, 9 h, 12 h, and 24 h after surgery. The AUC was used to add temporality to the pain measure. Sufentanil consumption was measured as the number of PCIA boluses administered from 0 to 24 h postoperatively in boluses, which will be extracted from the electronic PCIA pump records.

##### Secondary endpoints

1. Postoperative pain intensity measured by the NRS (at rest and on movement) at 0.5, 3 h, 6 h, 9h, 12 h, 24 h, 36 h and 48 h postoperatively.
2. Cumulative opioid consumption at 0.5, 3 h, 6 h, 9 h, 12 h, 24 h, 36 h, and 48 h postoperatively expressed as the administered boluses from the PCIA pump (each bolus is 1 ml and contains 0.04 μg/kg sufentanil).
3. Intraoperative anesthetic drug dosage.
4. Intraoperative hemodynamic parameters at incision, at exposure of the vertebral plate, before and after rod placement, and at wound closure completion.
5. Time to first rescue medication and dose of rescue medication.
6. Overall incidence of intraoperative and postoperative complications, including the following:
  - analgesic complications, such as wound infection, hematoma, and neurological complications.
  - Opioid-related side effects, including postoperative nausea and vomiting (PONV), mental status changes/hallucinations, sedation, and respiratory distress.
  - Surgical complications, such as incision infection, subcutaneous emphysema, hardware failure/implant removal, wound dehiscence, pseudarthrosis, hemothorax, substantial heterogeneity, pneumothorax, pleural effusion, cerebrospinal fluid leakage, spinal cord damage.
7. Quality of recovery, including the following:
  - Time to first mobilization.
  - Time to first flatus (hours); bowel movement (hours); liquid ingestion (hours); and solid-food ingestion (hours).
  - Quality of recovery measured by the Quality of Recovery-15 (QoR-15) score on the day on postoperative days (POD) 1 and 2 [27].
  - Length of hospital stay (days).
8. Total cost.

### Data collection

Patient demographics (age, sex, body mass index), ASA status, comorbidities, number of fused levels, and Cobb’s angle will be collected. The following intraoperative data will be collected: duration of surgery (form incision to closure), duration of anesthesia (from induction to extubation), intraoperative medications, blood loss and transfusion, and fluid balance. Outcome variable will be recorded according to the follow-up plan. Case report forms will be used to collect data and will be kept timely, correctly, and securely, and monitored by the ethics committee of our institution, and the data monitoring committee every year, which is set up by Beijing Children’s Hospital with members have no interest relationship with the study. The process will be independent from investigators. Only authorized researchers will have access to the final trial dataset.

### Statistical analysis

#### Sample size calculation

To determine the sample size for the primary outcome, we treated the NRS score as a continuous variable. Our pilot investigation showed the AUC of 24 h-NRS score of 82 ± 21 at 24 h after surgery for the control group and 63 ± 25 for the ESPB group. With a 5% significance level and a power of 90%, we calculated that each group should contain at least 33 patients. In the pilot study, the number of cumulative 24-h PCIA boluses was 25 ± 6 for patients in the control group and 18 ± 9 in the ESPB group. With a significance level of 0.05 and a power of 90%, 27 patients were estimated per group. The larger sample size of 33 patients in each group was chosen, which we increased to 37 to allow for 10% dropouts. The sample size was estimated by PASS software (version 15.0; NCSS PASS, UT, USA).

#### Endpoint analysis

The primary joint outcome was the AUC of NRS score at rest over time and opioid consumption measured by the number of PCIA boluses in postoperative 24 h. We defined the between-group difference of the primary analysis to be significant only when the difference of NRS and PCIA boluses are both associated with *p* < 0.05. Otherwise, the presence of *p* > 0.05 on any outcome indicated unsignificant pain relief of ESPB compared to the control group. An independent two-sample *t* test or Mann‒Whitney *U* test will be used based on the distribution of variables. The NRS scores and PCIA boluses up to 48 h after surgery and the intraoperative hemodynamics at different time points will be analyzed using repeated-measures two-factor analysis of variance (ANOVA) with a Bonferroni correction or one-way ANOVA as well as a generalized estimated equation (GEE) model as appropriate. For baseline characteristics and other continuous outcomes measured at one time point, two-sample *t* tests or Mann‒Whitney *U* tests will be performed. Episodes of complications will be analyzed by the chi-square test or Fisher’s exact test. The primary analyses will be conducted by intention-to-treat, and sensitivity analysis will be performed on a per-protocol basis.

##### Descriptive and analytical statistics

Continuous data are presented as the mean (± standard deviation (SD)) or median (interquartile range (IQR)) and analyzed by two-sample *t* test or Mann‒Whitney *U* test based on the distribution of variables, as determined by the Shapiro–Wilk test. Categorical data will be reported as numbers (percentages) and compared by the chi-square test or Fisher’s exact test. A two-tailed *P* value less than 0.05 will be regarded as statistically significant. The statistical analyses will be performed with the SPSS 25.0 statistical package (IBM SPSS Inc., Chicago, IL, USA).

#### Missing values

Patients with missing data on the primary outcomes will be excluded from analysis. For the baseline variables or secondary outcomes, missing data will not be replaced. Data from patients with missing values on primary outcomes will be analyzed in sensitive analysis, and missing values will be imputed with the median for each respective time point.

### Safety considerations

The researchers will adhere to the operation protocol strictly to minimize the risk of adverse events (AEs) and severe adverse events (SAEs). Medications are prepared and double-checked by specialized nurses. The operation of ESPB will be conducted by a fixed and experienced team under the extensive monitoring of vital signs to avoid AEs such as local anesthetic systemic toxicity. Experts are ready to provide immediate support if needed. For participants who are harmed during the investigation, the cost of treatment will be borne, and financial compensation will be paid in accordance with the relevant national regulations and laws.

### Oversight and monitoring

The ethics committee and data monitoring committee are appointed to oversight and monitor the project and provide advice on all aspects of the study. The PI will oversee the operational issues of the project. The whole research team will meet every month to check the progress of the trial. The data of AEs will be collected and recorded. Severe adverse events (SAEs) will be reported to the ethics committee and data monitoring committee, who will meet every 2 months to ensure the safety of the trial.

All relevant parties, including the investigators, IRB, trial registries, site staff, and other relevant authorities, will be notified of any modifications to the protocol during the entire trial period. The participants will be informed of relevant changes to the protocol as soon as possible or immediately in the event of major safety or efficacy concerns.

## Discussion

Typically, as the standard procedure for surgical management of AIS, PSF involves three to eight levels of spinal exposure, massive blood loss, and extensive muscle dissection, which causes severe postoperative pain,^28^ thus requiring high opiate usage [29,30]. In addition, because of the effect of sevoflurane on intraoperative neurophysiological monitoring (IONM) of movement, the large use of intraoperative opioids, usually remifentanil, seems to be necessary but may be associated with hyperalgesia in the postoperative period, further leading to a postoperative increase in opioids [31], and an increased risk of complications [32]. Physicians have taken an effort to decrease opioid prescriptions by using multimodal analgesia approaches, including neuraxial or regional techniques, in the postoperative period [33]. ESPB has earned its efficacy and reliability in adult patients undergoing lumbar spine surgeries [34], cervical spine surgeries [35], and lumbosacral spine [11,36] and thoracic spine surgeries [10]. Recent studies have even reported that ESPB shows more reliable spread and cover to the dorsal rami than to the ventral rami [37]. If it can be used for scoliosis surgery, there will be several advantages: it has no effect on IONM, provides stable hemodynamics, reduces blood loss, and, most importantly, reduces the pain and the use of opioids.

ESPB are feasible following our protocol. A single injection can block spinal nerves at multiple levels because of the extensive spread of LA in the musculofascial plane [9]. The dermatomal distribution of sensory loss by a single injection can cover the area from the parasternal to the midline of the lower back [38]. Because the number of segments invaded by idiopathic scoliosis is often greater and the lower thoracic segment ESPB may only spread to the L2–L3 level [37], we adhere to the following rule when choosing the plane of injection of ESPB: the midpoint of the involved segments (planned incision) is chosen in cases involving ≤ 5 segments. For example, for children undergoing fusion of L1−5 segments, we choose to make the injection at the L3 level, where the ESPB using 0.3% ropivacaine 0.5 ml/kg can subsequently diffuse to the L1−L5 levels to meet the surgical needs [39]. If fusion is planned for ≥ 6 segments, the incision is divided into two segments, and the midpoint of each segment is chosen as the injection site [24]. However, there are no pharmacokinetic data or solid conclusions on the maximal safe concentration and volume for ESPB so far [40]. The literature regarding pediatric use is even more limited. The volumes used in both our institution and previous publications are 0.3–0.5 mL/kg per side, with LA concentrations of 0.2% to 0.25% for infants and children, and 0.5% for adolescents [25]. However, it is imperative to emphasize that, to date, no pharmacokinetic data for ESPBs are available. This further reinforces the need to evaluate the safe maximal doses of concentration and volume for the age range [41].

In addition, the postoperative analgesia effect was illustrated by analyzing both the NRS score and PCIA boluses of opioids jointly in our study, since neither of the two ubiquitous outcomes of efficacy in trials of analgesics measured pain adequately when assessed alone [42]. The AUC-NRS adds a temporal dimension to the pain measurement, outbalances uneven measurement intervals, provides an integration of pain intensity versus time, and characterizes the trajectory of postoperative pain and analgesic drug efficacy more comprehensively. Compared with analyzing the discrete NRS score separately, this method increases statistical strength due to decreasing the risk of type 1 error in multiple significance tests. Additionally, the AUC-NRS is commensurable with opioid consumption, as it can fit along identical time intervals to better reflect the burden of pain over time [43].

However, no consensus has been reached regarding universal methods and the most appropriate statistical methods pertaining to outcome estimates in acute pain research. When applying the AUC-NRS method, potential erroneous interpolated estimates of pain during assessment intervals may occur. Additionally, clinically important indices such as the time to recurrence of pain, maximal pain experienced, and number of breakthrough pain episodes are not addressed.

In conclusion, pain management is a crucial aspect in the recovery of pediatric patients undergoing open spine surgery. To our knowledge, this study is the first to evaluate ESPB regarding efficacy in pediatric AIS patients undergoing PSF. The expected results of our study might prove the potential of ESPB to be recommended for perioperative analgesia in major open spine surgery.

## Trial status

The study is at the patient enrollment and data collection stage currently. The version of this study protocol is version 1.1 and was approved on 16 July 2023. Patient recruitment started on 1 September 2023 and is expected to be finished by 31 July 2024.

## Supplementary Information

Additional file 1. SPIRIT Checklist.

Additional file 2. Informed consent.

## Data Availability

The data including the full protocol, participant-level dataset, and statistical code will be available from the corresponding author upon request.

## Abbreviations

AIS: Adolescent idiopathic scoliosis
ASA: American Society of Anesthesiologists
AUC: Area under the curve
ESPB: Erector spinae plane block
IONM: Intraoperative neurophysiological monitoring
LA: Local anesthetic
NRS: Numerical rating scale
PACU: Post anesthesia care unit
PCIA: Parent-controlled intravenous analgesia
POD: Postoperative days
PONV: Postoperative nausea and vomiting
PSF: Posterior spinal fusion
QoR-15: Quality of Recovery-15 score
