# Supplementary material for: The effect of bilateral ultrasound-guided erector spinae plane block on postoperative pain control in idiopathic scoliosis patients undergoing posterior spine fusion surgery: study protocol of a randomized controlled trial
Source: Trials. 2024 Jul 22;25:498. doi: 10.1186/s13063-024-08331-2 (PMC11265167; doi:10.1186/s13063-024-08331-2)
Supplement: Supplementary file 2 — Additional file 2. Informed consent. [file 13063_2024_8331_MOESM2_ESM.docx]

**Informed page**

**Dear children and parents:**

We invite you to participate in the clinical study titled “The effect of bilateral ultrasound-guided erector spinae plane block on postoperative pain control in idiopathic scoliosis patients undergoing posterior spine fusion surgery”, which was approved by the Beijing Children's Hospital of Capital Medical University. This study complies with the Declaration of Helsinki and the principles of clinical trial quality management standards and has passed the review of the Ethics Committee of Beijing Children's Hospital affiliated with Capital Medical University.

Before you decide whether your child will participate in this study, please read the following as carefully as possible. It can help you understand why and how to participate in the research, the procedures and duration, and the benefits, risks and discomforts associate with the research. If you do not fully understand the content described in this consent form, you can consult the researcher or doctor to acquire a detailed explanation. This study is only available if both you and your child agree to participate. Your child's benefits and treatment will not be affected in any way if he or she does not participate in the study. You may also withdraw at any time during the study. If you decide to participate, you must sign this consent form, which confirms that you agree to take part in the study and allow the researcher to use your child's medical information.

**1. Why do we carry out this research?**

Postoperative pain after scoliosis surgery is severe, affecting the rehabilitation and physical and mental health of children. It is often difficult to achieve satisfactory analgesia effects with intravenous analgesia or oral medication alone. Erector spinal plane block (ESPB) is easy to perform, has low risk, and provides a good analgesic effect in various surgeries, but its effect on pediatric scoliosis patients remains controversial. The aim of this study was to evaluate the effect of ultrasound-guided bilateral erector spinae plane block combined with a multimodal analgesic program for postoperative analgesia in children and adolescents undergoing scoliosis surgery.

**2. How many people will participate in the study subjects?**

Seventy-four subjects will participate in this study.

**3. How was this research carried out?**

If you and your child agree to participate in this study, your child will undergo the following checks to further confirm his or her suitability for participating in this study:

• Physical examination and medical history inquiry,

• Important physical signs (such as breathing, body temperature, heartbeat, etc.),

• Blood testing,

• ECG, echocardiogram, chest radiograph, etc.

All of the above tests are part of our routine procedures and are required for all children undergoing surgery. The final decision as to whether your child can participate in the study will be made after the investigator has examined him or her.

The inclusion criteria were as follows:

(1) Aged younger than 18 years and undergoing elective PIS;

(2) American Society of Anesthesiologists (ASA) physical status of I to II.

The exclusion criteria were as follows:

(1) Severe comorbidities such as cardiac insufficiency and liver or renal dysfunction;

(2) Contraindications to ESPB as follows: coagulation abnormalities, hemorrhagic diseases, puncture site infections, and preexisting neurological deficits;

(3) Allergy to LA or other study medication;

(4) Chronic pain characterized by preoperative opioid use for more than 3 months;

(5) Cognitive impairment or mental illness rendering the patient unable to cooperate in the pain assessment;

(6) Inability of the guardian to use a postoperative analgesia pump.

Participants were randomly divided into an erector spinal muscle plane block group or a control group by computer, and each participant had an equal chance of being assigned to each group. Neither you nor your doctor can choose your treatment group. Throughout the study, we will follow up on the health status of the participants. When the study is over, we will write a report of the results, but we will not use personal information such as your child's name in the article.

**4. If I participate in the study, what should I do?**

If you are willing to participate in this study, you should follow your doctor's instructions, learn how to use the analgesia pump, and cooperate with the study visits, which will include bedside follow-up during the hospitalization period and telephone follow-up at approximately 3 months postoperatively. Follow-up visits during the hospitalization period will include the child's pain score, time of eating and drinking, bowel movements, time of getting out of bed, nausea, vomiting, fever, postoperative complications, and other health conditions. Follow-up visits at 3 months after surgery will include status of the child's recovery and whether he or she is taking oral analgesic medication.

**5. Do I have other treatment options?**

If you do not participate in this study, your child will receive routine postoperative constant-rate IV analgesic pump analgesia, and all other medical regimens will be based on your child's condition and will not be affected by your decision regarding participation in the study. In addition, you may withdraw from the study at any time without losing any of the benefits you would otherwise receive. If you choose to withdraw, we will continue to administer routine analgesia to your child. However, due to safety concerns, it is possible that we may perform some relevant medical tests after you make the decision to withdraw, all of which are noninvasive and will not cause discomfort or pain.

**6. What are the risks of participating in the research?**

All medical procedures have the potential for side effects or adverse reactions. Electronic pump analgesia may be associated with mechanical failure and adverse reactions to analgesic medications such as nausea, vomiting, and respiratory depression. Nerve block is an invasive operation, and puncture-related complications may occur, including bleeding at the puncture site, hematoma, infection, pneumothorax, and local anesthetic drug poisoning. In the case of any of the above situations, we will strictly adhere to the standard operating procedures of our institution to minimize the risk of adverse events that might be caused by epidural or regional nerve block. All the operations will be conducted under extensive monitoring of vital signs and the direct presence of experts in the field who are able to provide immediate support if needed. In addition, any treatment may be ineffective. Cases of poor analgesia may occur.

**7. What are the benefits of participating in the research?**

This study may or may not lead to a decrease in your child's postoperative pain level and accelerated recovery, or it may not improve your child's postoperative pain or prognosis. However, this study will improve postoperative analgesia programs and comfort medicine for pediatric scoliosis patients, address the current lack of pediatric analgesia programs, and promote the application and dissemination of the erector spinae block technique so that more children and families can benefit.

**8. Do I need to pay related fees to participate in the research?**

The regular cost of general anesthesia, nerve block puncture needles, and local anesthetics is the responsibility of the subjects themselves.

**9. What happens if I get damaged while participating in the study?**

If your health suffers from research-related damage or injury due to participation in this research, please notify the research doctor immediately, and they will be responsible for taking appropriate treatment measures. If damage is caused by the research, the cost of treatment will be borne, and financial compensation will be paid in accordance with the relevant national regulations and laws. Even if you have provided written informed consent, you still retain all your legal rights.

**10. Is my personal information confidential?**

Yes. Your medical records will be kept in the hospital. Only the investigators, research authorities, and ethics committees will be allowed to access your medical records. Any public report on the results of this research will not disclose your personal identity. We will make every effort to protect the privacy of your personal medical information within the scope permitted by law.

**11. Do I have to participate in the research?**

No. Participation in this research is completely voluntary. You can refuse to participate in the research or withdraw at any time during the research process for any reason. This decision will not affect the doctor's treatment.

**12. How will participating in the study affect my life?**

You may feel that follow-up and visits from researchers during hospitalization are inconvenient. Some tests may make you feel a little bit uncomfortable. In addition, during the entire study period, you can no longer participate in any other clinical studies related to drugs or medical devices.

**13. Related Consultation**

If you have any questions related to this research, please contact Yi Ren, 59616453. If you have any questions related to your own rights or if you want to report your dissatisfaction and concerns during the process of participating in this research, please contact the Ethics Committee of Beijing Children's Hospital: 59616644, bch_irb@163.com.

**Consent signature page**

**Subject statement:**

**I have read the above introduction to this research, and I voluntarily participate in this research.**

**I □ agree or □ disagree with the use of my medical records and examination specimens in studies other than this one.**

**I fully understand:**

**1. the risks and benefits that may arise from participating in this research.**

**2. that I can consult the doctor for more information at any time.**

**3. that my child can withdraw from this study at any time without discrimination or retaliation, and medical treatment and rights will not be affected.**

Subject's name:

Subject’s signature:

Date:

Signature of guardian or legal representative:

Contact number:

Date:

**Investigator's statement:**

**I confirm that I have explained the details of this research to the subjects, especially the possible risks and benefits of participating in this research.**

Investigator’s signature:

Contact number:

Date:
